# Rapid Weight Loss Habits before a Competition in Sambo Athletes

**DOI:** 10.3390/nu13041063

**Published:** 2021-03-25

**Authors:** Flavia Figlioli, Antonino Bianco, Ewan Thomas, Valdemar Stajer, Darinka Korovljev, Tatjana Trivic, Nebojsa Maksimovic, Patrik Drid

**Affiliations:** 1 Sport and Exercise Sciences Research Unit, University of Palermo, 90144 Palermo, Italy; flaviafiglioli@hotmail.it (F.F.); antonino.bianco@unipa.it (A.B.); ewan.thomas@unipa.it (E.T.); 2 Faculty of Sport and Physical Education, University of Novi Sad, 21000 Novi Sad, Serbia; stajervaldemar@yahoo.com (V.S.); korovljev.darinka@gmail.com (D.K.); ttrivic@yahoo.com (T.T.); nebojsam@uns.ac.rs (N.M.)

**Keywords:** weight class, combat sports, rapid weight loss

## Abstract

Background: Like other combat sports, sambo has competition rules that divide athletes into categories based on gender, age and weight. Athletes in combat sports often resort to rapid weight loss (RWL) methods to be more competitive in lower weight categories and gain an advantage against lighter, smaller and weaker competitors. The aim of this study was to examine the methodology implemented by two different sambo age categories, junior and senior athletes, in order to attain RWL. Methods: The sample consisted of 103 male sambo elite athletes (seniors/juniors: age 28.5 ± 4.3/18.9 ± 0.8; height (m): 1.7 ± 0.1/1.8 ± 0.1; weight (kg): 76.3 ± 17.8/74.4 ± 16.3; BMI (kg/m^2^): 25.0 ± 3.8/23.7 ± 3.9) who completed a survey on RWL. Results: Athletes reported losing a mean of 5 kg starting approximately 12 days before a competition. The most common methodology reported by senior and junior sambo athletes was gradually increasing dieting, followed by sauna and plastic suit training. Less common methods adopted were laxatives, diuretics, the use of diet pills and vomiting. There were significant group differences for sauna and diet pill ingestion. Coaches and parents are influential people in the lives of athletes concerning the weight loss strategy to be adopted. Conclusions: This study’s results unequivocally confirm the prevalent practice of RWL in both senior and junior sambo athletes. Although athletes prevalently chose “less harmful” methods, there is a need to inform parents and coaches of the risks and benefits of RWL.

## 1. Introduction

The term “SAMBO” is the acronym of “САМозащита Без Оружия”, a phrase of Russian origin that means “self-defense without weapons”. Soviet Union troops used this combat sport as a training tool [1]. From a fighting method, sambo became an officially recognized combat sport in the 1940s, as declared by the Committee of Sports Union of Soviet Socialist Republics. After 80 years of growth and development, sambo has obtained worldwide recognition and has been recently temporarily accepted by the International Olympic Committee [2]. In order to allow a more even participation, as for other combat sports, specific weight categories based on gender and age have also been adopted in sambo [3]. Nowadays, sambo has age classes ranging from cadet (14–15–16), youth (16–17–18 years old) and juniors (18–19–20 years old) to seniors (over 18 years old).

Many combat sports athletes try to reduce their weight because of competitive and tactical advantages or psychological reasons, such as increased self-esteem, pleasure and wellness. This turmoil of positive psychophysical feelings in the athlete often turns into rapid weight loss (RWL). Due to these practices, muscular injuries or postural imbalances may occur, which would require appropriate evaluation techniques to early detect biomechanical alterations [4,5].

In this article, particular attention is dedicated to the “cutting weight” issue widely discussed in the literature and demonstrated in weight category sports [6,7]. Rapid weight loss (RWL) can be achieved by different methods and behaviors (active or passive) and also due to influences from an external person that can encourage or teach ways to lose weight [7]. Regardless of the type of combat sport, methods of inducing RWL are very similar, often starting by reduced ingestion of fluids, fasting, skipping meals combined with supplementations, high training levels, plastic suit training and sauna use [7,8,9,10]. Considering that sambo is a contact sport, the number of health injuries due to RWL may concern the sport’s medicine area.

The present study’s aim is to compare junior and senior sambo athletes regarding the strategies adopted for rapid weight loss before a competition.

## 2. Materials and Methods

### 2.1. Participants

**Figure 1:**
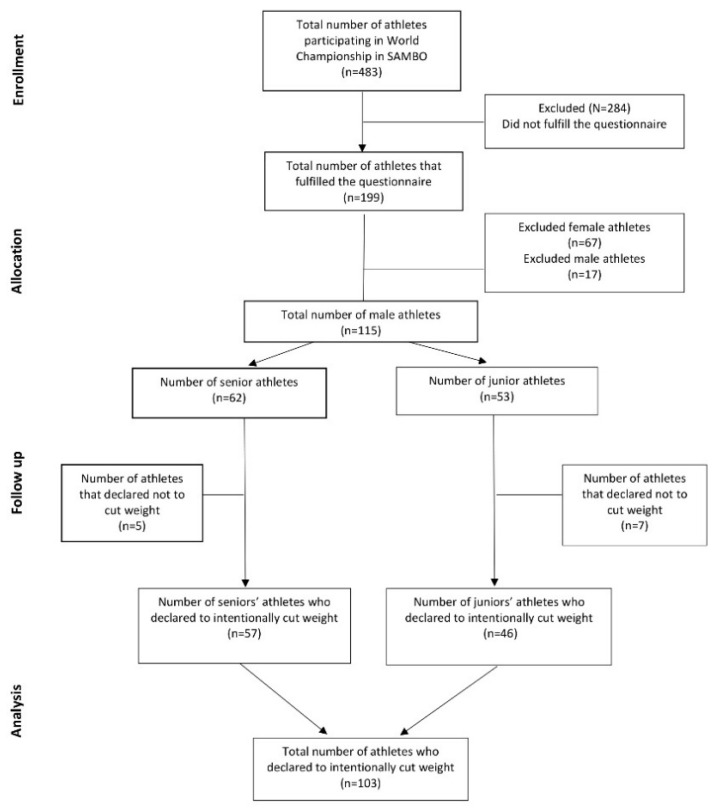
Flow of participants.

Athletes (*n* = 483) competing in the World Sambo Championship 2020 (juniors and seniors), coming from 35 different countries, were asked to voluntarily fill out an anonymous questionnaire regarding their RWL strategies. A total of 199 athletes (male = 132 and female = 67) agreed to participate in the survey. For the purpose of this study, only male athletes were considered. Of the 132 screened male athletes, 17 were excluded for incorrect questionnaire compilation. Of the 115 remaining athletes, 103 met the inclusion criteria by declaring that they intentionally cut their weight prior to competitions. The flow of participants in the study is presented in Figure 1.

The questionnaire by Artioli et al. [11] was adopted, consisting of questions regarding personal information, competitive level, weight, diet history and behaviors. All procedures received the university ethics committee’s approval to maintain the confidentiality and anonymity of the athletes’ responses. The main characteristics of the sambo competitors are shown in Table 1.

### 2.2. Data Assessment

Data were collected through a standardized questionnaire, with the aim to examine weight reduction history and weight loss methods adopted by the screened athletes and to investigate if someone’s suggestion influenced RWL strategies. The questions related to weight loss methods included the most commonly adopted strategies [6]. The questions regarding weight reduction history included the amount of weight lost before competitions, the mean period spent prior to the competitions engaged in weight loss, the amount of weight regained following the competition and the age at which athletes started adopting weight loss strategies. The frequency at which these strategies were adopted was subsequently classified as (1) always, (2) sometimes, (3) almost never, (4) never used and (5) not used anymore. Only categories 1 and 2 were considered as “adopted” for the weight loss strategy method. The main source of influence regarding RWL was screened by considering the following categories: teammate, fellow sambist, physician, personal trainer, coach, parents and dietitian. The frequency reported by the athletes regarding the source of influence was categorized as follows: (1) Not influential, (2) Little influential, (3) Unsure, (4) Somewhat influential and (5) Very influential. Only categories 4 and 5 were acknowledged as “influential” and, therefore, considered for the analysis.

### 2.3. Statistical Analysis

Data were analyzed through the SPSS statistical software (ver. 23.0). Data are presented as means and standard deviations. All data were checked for normality. Non-parametric analyses (chi-square test for cross-tabulation and Friedman’s test for analysis of interactions) were adopted when appropriate. Significance was set at *p* < 0.05.

## 3. Results

Regarding the total number of athletes who endorsed adopting RWL practices, 89.6% of participants admitted to have been intentionally cutting their weight before competitions. The mean age reported for the first RWL was 17.6 ± 4.7 years for seniors and 14.0 ± 3.9 for juniors. The seniors intentionally cut on average 5.7 ± 7.2 kg of body weight compared to juniors with 4.1 ± 2.1 kg. The senior and junior athletes stated that they started cutting body weight 12.4 ± 12.1 and 10.8 ± 7.5 days prior to the competition, respectively (Table 2). The analysis of interactions between groups highlights a significant effect only for question “e” (*p* < 0.001), indicating that junior athletes started adopting RWL practices earlier in life.

Concerning the methods used to achieve RWL, the most common was gradual dieting (77.7%), followed by sauna (74.8%), training with plastic suits (68%), skipping meals (66%), training in a heated room (60.2%), restricting fluid ingestion (57.3%), increased exercise (52.4%), spitting (50.5%), fasting (39.8%) and the use of a plastic suit all day (37.9%). Less common methods adopted were the use of laxatives (19.4%), diuretics (13.6%), diet pills (11.7%) and vomiting (7.8%) (Table 3). Concerning group differences in the methods used for weight loss, the chi-square tests showed significant differences for sauna use (*p* < 0.00) and diet pill ingestion (*p* < 0.02).

Putting together seniors and juniors showed that they were somewhat or very influenced by their coach (54.4%) followed by their teammates (28.2%). The least influential to athletes were dietitians and physicians (17.5% and 16.5%) (Table 4). Chi-square tests revealed statistically significant differences between seniors and juniors in the influence of parents (*p* < 0.04), where junior athletes were more often somewhat or very influenced by parents (45.7%) in contrast to senior athletes (19.3%).

## 4. Discussion

This study aimed to outline the methodologies adopted by sambo athletes in order to attain RWL before a competition. This study shows that most athletes, both seniors and juniors, adopted rapid weight loss practices before a competition and that these are mainly related to gradual dieting, use of sauna sessions, training in plastic suits and skipping meals. Concerning group differences regarding the RWL methods employed, these were present for sauna and diet pills. According to Barley et al. [12], who analyzed weight loss strategies in different combat sports, the most common strategies adopted were related to diet, fluid restriction and exercise. Furthermore, data from professional wrestlers and elite kickboxers highlight that reduced caloric intake, increasing exercise intensity, fluid restriction and training in plastic suits are frequently adopted practices [13,14]. These findings are in line with our results, underscoring the more frequent use of diet- and exercise-related weight loss strategies regardless of the combat sport taken into account. However, very different percentages were observed for other methods, such as sauna use and skipping meals [12], which seem to be more frequently adopted in sambo athletes.

Although weight loss practices such as vomiting, taking pills and diuretics have not been frequently reported in the literature, there is still concern regarding the relatively high frequency described among this population. Similar values (21.3%) were published by Berkovich et al. [15], who assessed weight loss habits in judo athletes.

Since no difference was reported among these practices across age groups, weight-control practices should be discouraged, especially in younger categories [16,17,18].

The results concerning the person who most influences the choice of RWL strategies show a significant contribution of parents for junior athletes, while this appeared to be one of the least influential categories of people for seniors. In contrast, coaches were the most influential people for senior athletes. Experienced athletes also relied on colleagues’ suggestions and previous experiences. These results were somewhat expected. Different authors have reported similar results [15,19,20,21], despite considering combat sports other than sambo. Very low percentages of athletes admit to having consulted a physician or a dietician regarding weight loss. As reported by a study considering Mixed Martial Arts athletes, a dietitian’s guidance may reduce the likelihood of unsafe RWL practices [22].

Another important aspect which needs to be stressed is the age at which these strategies are usually employed. We were able to identify a substantial difference between seniors and juniors, with the latter reporting having started RWL practices at the age of 14 compared to seniors (17 years of age). Interestingly, an investigation carried out in elite male kickboxers [14], who have an age very similar to our senior athletes, reported onset of RWL practices around the age of 18 years, which almost overlaps with the age declared by our senior athletes. Because of these disturbing results, there is a need for permanent education about RWL consequences and potential hazards conferred by important individuals who may influence young athletes, such as parents and coaches.

This study is not without limitations since our investigation cannot reveal if the athletes were sincere regarding the adopted practices. Another limitation is the methodology adopted. A cross-sectional investigation was carried out with information collected by the RWL questionnaire; therefore, it is impossible to evaluate physiological parameters or link health status to the weight loss or the practices adopted. However, this investigation allows us to understand the habits of sambo athletes competing at an international level from countries worldwide, representing an interesting perspective for a general understanding of RWL practices in sambo competitors.

## 5. Conclusions

Rapid pre-competition weight loss habits among sambo athletes highlighted that almost every athlete adopts these practices before a competition. The most common strategies such as dieting, increasing exercise intensity or using a sauna seem prevalent compared to other habits such as vomiting or taking pills and laxatives. However, a still worrying percentage have endorsed these latter methods before competitions. Since parents and coaches seem to be the primary sources of influence regarding weight loss practices, there is a need to inform and educate them about the potential harmful effects of RWL in combat sport athletes.

## Figures and Tables

**Table 1:** Main characteristics of the sambo competitors.

Variable	Group	Mean ± SD
Age (yrs.)	Seniors	28.5 ± 4.3
	Juniors	18.9 ± 0.8
Height (m)	Seniors	1.7 ± 0.1
	Juniors	1.8 ± 0.1
Weight (kg)	Seniors	76.3 ± 17.8
	Juniors	74.4 ± 16.3
BMI (kg/m^2^)	Seniors	25.0 ± 3.8
	Juniors	23.7 ± 3.9
Age began practicing sambo (yrs.)	Seniors	12 ± 6.4
	Juniors	10.2 ± 3.2
Age began competing sambo (yrs.)	Seniors	14 ± 6.3
	Juniors	11.1 ± 3.2

BMI—body mass index; SD—standard deviation.

**Table 2:** Weight reduction history reported by two categories sambo competitors.

Questions	Group	Mean	SD	χ^2^	*p*
a. What is the most weight you have cut to compete in your career? (kg)	Seniors	8.5	9.3	0.10	0.74
	Juniors	7.4	3.5		
b. How many times did you cut weight to compete last season? (number of times)	Seniors	3.1	2.1	0.16	0.69
	Juniors	3.4	2.5		
c. How much weight do you usually cut before the competition? (kg)	Seniors	5.7	7.2	2.65	0.10
	Juniors	4.1	2.1		
d. How many days before a competition do you usually cut weight? (days)	Seniors	12.4	12.1	0.01	0.92
	Juniors	10.8	7.5		
e. At what age did you start to cut weight before a competition? (yrs.)	Seniors	17.6	4.7	29.8	0.00 *
	Juniors	14.0	3.9		
f. How much weight do you usually regain after a competition? (kg)	Seniors	4.6	2.6	2.57	0.11
	Juniors	4.1	2.6		

SD—standard deviation; χ^2^—Friedman test; *p*—probability; *—significant.

**Table 3:** Frequency distribution for weight loss methods reported by two categories of sambo competitors.

Methods	Group	Always	Sometimes	Almost Never	Never Used	Do Not Use Any More	χ^2^	*p*
Gradual dieting	Seniors	31 (54.4%)	13 (22.8%)	3 (5.3%)	5 (8.8%)	5 (8.8%)	4.61	0.33
	Juniors	22 (47.8%)	14 (30.4%)	6 (13%)	3 (6.5%)	1 (2.2%)		
Skipping meals	Seniors	9 (15.8%)	28 (49.1%)	11 (19.3%)	7 (12.3%)	2 (3.5%)	0.92	1.00
	Juniors	7 (15.2%)	24 (52.2%)	8 (17.4%)	5 (10.9%)	2 (4.3%)		
Fasting	Seniors	4 (7%)	20 (35.1%)	12 (21.1%)	17 (29.8%)	4 (7%)	0.72	0.95
	Juniors	3 (6.5%)	14 (30.4%)	11 (23.9%)	13 (28.3%)	5 (10.9%)		
Restricting fluid ingestion	Seniors	12 (21.1%)	20 (35.1%)	13 (22.8%)	11 (19.3%)	1 (1.8%)	1.21	0.88
	Juniors	7 (15.2%)	20 (43.5%)	11 (23.9%)	7 (15.2%)	1 (2.2%)		
Increased exercise	Seniors	18 (31.6%)	14 (24.6%)	21 (36.8%)	2 (3.5%)	2 (3.5%)	7.43	0.12
	Juniors	16 (34.8%)	6 (13%)	17 (37%)	0 (0.0%)	7 (15.2%)		
Training in heated room	Seniors	13 (22.8%)	17 (29.8%)	15 (26.3%)	11 (19.3%)	1 (1.8%)	4.21	0.38
	Juniors	14 (30.4%)	18 (39.1%)	10 (21.7%)	4 (8.7%)	0 (0.0%)		
Sauna	Seniors	13 (22.8%)	27 (47.4%)	4 (7%)	10 (17.5%)	3 (5.3%)	19.72	0.00 *
	Juniors	22 (47.8%)	15 (32.6%)	9 (19.6%)	0 (0.0%)	0 (0.0%)		
Training in plastic suits	Seniors	18 (31.6%)	20 (35.1%)	14 (24.6%)	5 (8.8%)	0 (0.0%)	1.60	0.81
	Juniors	16 (34.8%)	16 (34.8%)	9 (19.6%)	4 (8.7%)	1 (2.2%)		
Use plastic suit all day	Seniors	7 (12.3%)	11 (19.3%)	15 (26.3%)	23 (40.4%)	1 (1.8%)	3.92	0.42
	Juniors	10 (21.7%)	11 (23.9%)	7 (15.2%)	18 (39.1%)	0 (0.0%)		
Spitting	Seniors	6 (10.5%)	20 (35.1%)	6 (10.5%)	23 (40.4%)	2 (3.5%)	6.12	0.19
	Juniors	4 (8.7%)	22 (47.8%)	9 (19.6%)	9 (19.6%)	2 (4.3%)		
Laxative	Seniors	2 (3.5%)	6 (10.5%)	6 (10.5%)	43 (75.4%)	0 (0.0%)	6.08	0.19
	Juniors	2 (4.3%)	10 (21.7%)	6 (13%)	26 (56.5%)	2 (4.3%)		
Diuretic	Seniors	1 (1.8%)	4 (7%)	5 (8.8%)	46 (80.7%)	1 (1.8%)	4.45	0.35
	Juniors	2 (4.3%)	7 (15.2%)	3 (6.5%)	31 (67.4%)	3 (6.5%)		
Diet pills	Seniors	0 (0.0%)	4 (7%)	4 (7%)	49 (86%)	0 (0.0%)	9.91	0.04 *
	Juniors	4 (8.7%)	4 (8.7%)	6 (13%)	30 (65.2%)	2 (4.3%)		
Vomiting	Seniors	0 (0.0%)	3 (5.3%)	2 (3.5%)	49 (86%)	3 (5.3%)	6.95	0.14
	Juniors	1 (2.2%)	4 (8.7%)	7 (15.2%)	33 (71.7%)	1 (2.2%)		

χ^2^—chi-square test; *p*—probability; *—significant.

**Table 4:** Frequency distribution for people influencing rapid weight loss (RWL).

Influence	Group	Not Influential	Little Influential	Unsure	Somewhat Influential	Very Influential	χ^2^	*p*
Teammate	Seniors	20 (35.1%)	11 (19.3%)	4 (7%)	11 (19.3%)	11 (19.3%)	7.37	0.12
	Juniors	13 (28.3%)	18 (39.1%)	2 (4.3%)	10 (21.7%)	3 (6.5%)		
Fellow sambist	Seniors	20 (35.1%)	13 (22.8%)	6 (10.5%)	8 (14%)	10 (17.5%)	7.84	0.10
	Juniors	13 (28.3%)	10 (21.7%)	7 (15.2%)	14 (30.4%)	2 (4.3%)		
Physician	Seniors	30 (52.6%)	9 (15.8%)	6 (10.5%)	9 (15.8%)	3 (5.3%)	3.44	0.50
	Juniors	26 (56.5%)	10 (21.7%)	5 (10.9%)	5 (10.9%)	0 (0%)		
Personal trainer	Seniors	25 (43.9%)	11 (19.3%)	5 (8.8%)	10 (17.5%)	6 (10.5%)	1.17	0.88
	Juniors	19 (41.3%)	11 (23.9%)	2 (4.3%)	8 (17.4%)	6 (13%)		
Coach	Seniors	14 (24.6%)	6 (10.5%)	8 (14%)	14 (24.6%)	15 (26.3%)	7.71	0.10
	Juniors	5 (10.9%)	11 (23.9%)	3 (6.5%)	10 (21.7%)	17 (37%)		
Parents	Seniors	34 (59.6%)	9 (15.8%)	4 (7%)	5 (8.8%)	5 (8.8%)	10.96	0.03 *
	Juniors	15 (32.6%)	8 (17.4%)	2 (4.3%)	11 (23.4%)	10 (21.7%)		
Dietitian	Seniors	34 (59.6%)	5 (8.8%)	7 (12.3%)	8 (14%)	3 (5.3%)	9.41	0.05
	Juniors	24 (52.2%)	13 (28.3%)	2 (4.3%)	3 (6.5%)	4 (8.7%)		

χ^2^—chi-square test; *p*—probability; *—significant.

## Data Availability

The data presented in this study are available on request from the corresponding author.
